# Understanding the history of the late Roman synagogue at Huqoq in Israel’s Galilee through radiocarbon dating and observations on site formation

**DOI:** 10.1371/journal.pone.0313512

**Published:** 2025-02-11

**Authors:** Dennis Mizzi, Daniel Schindler, Elisabetta Boaretto, Michael Chazan, Jodi Magness

**Affiliations:** 1 Department of Middle Eastern and Asian Languages and Cultures, University of Malta, Msida, Malta; 2 Independent Scholar, Minnesota, United States of America; 3 Scientific Archaeology Unit, D-REAMS Radiocarbon Laboratory, Weizmann Institute, Rehovot, Israel; 4 Department of Earth Science, University of Toronto, Toronto, Ontario, Canada; 5 Department of Religious Studies, University of North Carolina, Chapel Hill, North Carolina, United States of America; Israel Antiquities Authority, ISRAEL

## Abstract

Galilean-type synagogues are monumental, basilical structures found in northern Israel that have long been dated, mainly on stylistic grounds, to the 2nd – 3rd centuries CE. This chronology is influenced by historical considerations – specifically, the notion that monumental synagogues must have been constructed before Jews came under Christian rule in the early 4th century. However, the stratigraphic contexts of the pottery and coins associated with the foundation of these buildings suggest they represent an architectural innovation dating to the 4th (especially the later 4th) to 6th centuries CE. The Huqoq Excavation Project was initiated in 2011 with the goal of determining the construction date of a Galilean-type synagogue through controlled stratigraphic excavation, including the systematic collection of samples for radiocarbon dating along with micromorphological analysis of the fill deposits. Here we present the initial results of these analyses, which indicate that the radiocarbon ages conform with the pottery data to place the construction of the Huqoq synagogue in the late 4th – early 5th centuries (late Roman period). However, the ages of dated samples from thick fills overlying the floor, which were deposited when the synagogue was rebuilt and expanded in the early 14th century (late medieval/Mamluk period) do not reflect the stratigraphic sequence.

## Introduction

Galilean-type synagogues are monumental, basilical structures that have long been dated, mainly on stylistic criteria, to the 2nd – 3rd centuries CE. This chronology is influenced by historical considerations – specifically, the notion that monumental synagogues must have been constructed before Jews came under Christian rule in the early 4th century. However, the stratigraphic contexts of the pottery and coins associated with the foundation of these buildings suggest they represent an architectural innovation dating to the 4th (especially the later 4th) to 6th centuries CE [1,2]. Consequently, the appearance of monumental synagogue architecture in 4th century Palestine should be understood within the context of the rise of Christianity, which was legalized by Constantine in 313 CE, and the emergence of church construction. The results of our analysis also contradict a claim that Jewish settlement in Eastern Lower Galilee experienced a dramatic decline beginning in the mid-4th century [3–15].

The Huqoq Excavation Project was initiated in 2011 with the goal of determining the construction date of a Galilean-type synagogue through controlled stratigraphic excavation. From 2014 to 2023, the following institutions were consortium members of the Huqoq Excavation Project: The University of North Carolina at Chapel Hill; Brigham Young University; the University of Toronto; Austin College (TX) (2019-2023); Baylor University (2016-2019); and Trinity University (TX) (2014). Before the excavations commenced, fragments of stone columns and other architectural pieces scattered amid the rubble of the village of Yaquq/Yakuk (which was abandoned in 1948 and bulldozed in the mid-1960s) had pointed to the existence of a synagogue at the site, as they resemble architectural elements in Galilean-type synagogues at other sites. And indeed, the excavations have brought to light a monumental, Galilean-type synagogue paved with stunning mosaics depicting an array of biblical stories and non-biblical scenes and motifs [1]. In the 14th century (late medieval/Mamluk period) – hundreds of years after the synagogue’s abandonment – the structure was rebuilt on an even larger scale, apparently also for use as a synagogue [16].

From the beginning of excavations at Huqoq, the focus has been on the careful documentation of the stratigraphy to address questions of the chronology of Galilean-type synagogues. As part of this program, the collection of samples was undertaken for radiocarbon dating, along with micromorphological analysis of the fill deposits. Due to the precision of material culture chronologies for the Roman-Byzantine periods, and especially a reliance on numismatic evidence, radiocarbon dating is rarely used in these contexts in Levantine archaeology. Recent exceptions include research on garbage dumps outside Elusa, pigeon towers in the Negev, and a kiln from Tel Qatra [17–19]. The application of radiocarbon dating to monumental construction includes research on the West Church at Umm el-Jimal and Jacob’s Well in Nablus [20,21]. The integration of microarchaeology tools to characterize the contexts of radiocarbon samples into the development of chronologies for monumental structures improves precision and eliminates noisy results as has been demonstrated in the dating of the Gihon Tower and Wilson's Arch in Jerusalem [22,23]. We present here the initial results of chronological research at Huqoq with radiocarbon age determinations integrated with observations on material culture and site formation. Our results demonstrate a high probability that the Huqoq synagogue was built in the late 4th – early 5th centuries, but these results also point to some challenges that limit the precision of radiocarbon dates. A major problem, which relates to site formation processes, is one that is likely to apply to many contexts of monumental construction in which an initial structure undergoes reconstruction. Because the deposit overlying the mosaic floor is a fill that was used to raise the level of the floor in the late medieval period, the ages of dated samples from this deposit have no relation to the original (late Roman) synagogue’s period of use. However, these results also point to the potential of radiocarbon dating to contribute to an understanding of site formation processes.

### Site architecture and stratigraphy

**Fig 1:**
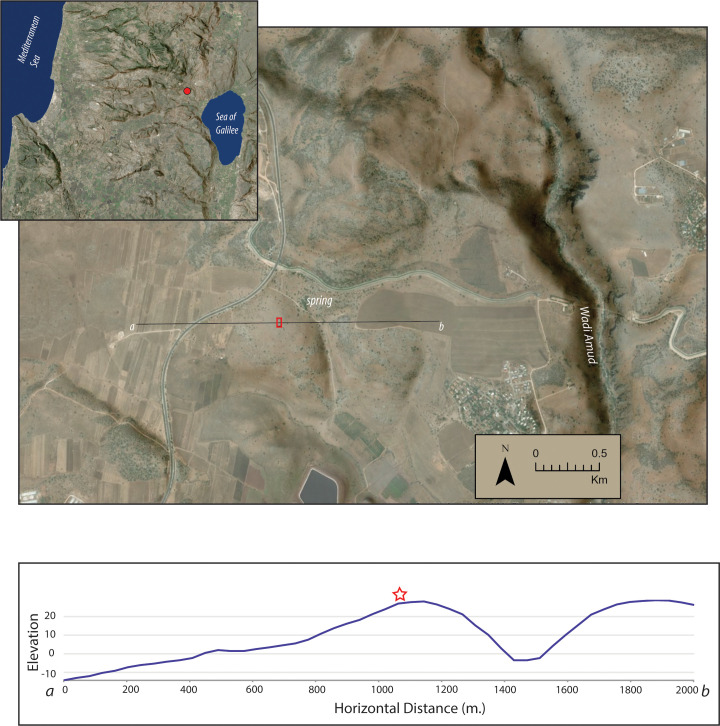
Image showing the geographic setting of Huqoq at the top of a low hill overlooking agricultural lands to the west and the location of a spring at the base of the hill to the east of the site. Below is a profile showing elevations along an east-west transect (black line on upper image). The red square in the upper image shows the position of the synagogue, and the red star shows the position of the synagogue in the lower image. Map created in ArcGIS Pro using Global Imagery (Clarity) and World Hillshade basemaps (source Esri, NASA, NGA, USGS). under terms of license to the University of Toronto.

Ḥorvat Ḥuqoq (henceforth Huqoq) is located in Eastern Lower Galilee, 3 km northwest of the Sea of Galilee and 12.5 km north of Tiberias. The remains of the ancient settlement (ca. 25 – 30 dunams) are covered partly by the ruins of the 19th–20th century village of Yaquq/Yakuk (ياقوق‎) (ca. 10 dunams), which was inhabited until 1948 [1]. The site is situated on the top of a low limestone hill (elevation 23 m. asl) overlooking agricultural land in a valley to the west as well as agricultural fields at a higher elevation to the east that run into the Wadi Amud (Fig 1). In the vicinity of Huqoq, terra rosa soils develop in valley floors while most of the hilltops and slopes have only shallow, if any, soil cover. A spring at the base of the eastern slope of the hill is still active and appears to be on an ancient secondary road network. It appears that the Roman-Byzantine and later occupation levels at Huqoq lie on top of a long sequence of occupation that forms a low tel at the top of a hill. The earliest artifacts found at the site are pottery and a mace head fragment dating to the Early Bronze Age, and substantial quantities of Hellenistic pottery have been found in soundings under the late Roman synagogue’s floor. By the late Hellenistic and Roman periods, Huqoq was a Jewish agricultural village, and the Jerusalem Talmud refers to locals gathering seeds from wild mustard plants (*y. Shev.* 9.1, 38c). By the medieval period, the final resting place of the biblical prophet Habakkuk was firmly linked to the site (which had become known as Yaquq/Yakuk), attracting Jewish and non-Jewish pilgrims.

#### Late Roman synagogue.

**Fig 2:**
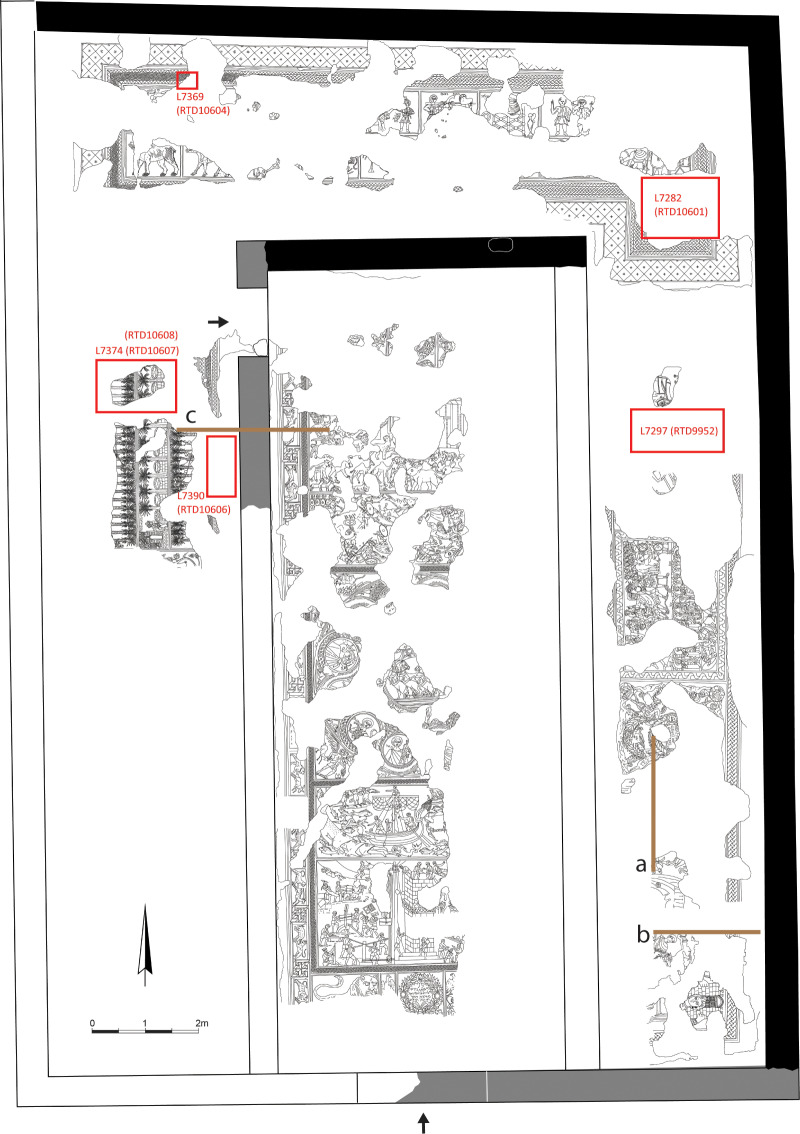
Plan of the late Roman synagogue at Huqoq at the end of the 2019 excavation season. The red boxes show the contexts for radiocarbon samples related to the laying of the mosaic floor or from deposits immediately overlying the mosaic floor. Brown lines indicate the position of the profiles shown in Fig 4. Note that the plan does not show the entrance in the east wall. Plan by Slava Pirsky, with adaptations by Dennis Mizzi and Michael Chazan.

The late Roman synagogue uncovered at Huqoq is a basilica, with the long walls on the east and west, entrance(s) in the south (Jerusalem-oriented) wall and in the east wall, and a stylobate that wrapped around the north, east, and west sides of the interior (Fig 2). The synagogue is 20 m long and 14.19 m wide. The nave is ca. 5 m wide and 0.20 m lower than the aisles, which are ca. 3.60 m wide. The synagogue’s interior was paved entirely with mosaics, which are divided into panels depicting an array of biblical stories and other motifs [1,24,25]. Soundings opened in areas where the mosaic floor was not preserved revealed that the mosaic was laid over a hard bedding of fine plaster with a smooth, flat surface that was laid over a rougher plaster layer including small pebbles and pieces of tesserae raw material, which in turn sat on a layer of basalt cobblestones embedded in a matrix of packed earth. Excavations below the north and west aisles of the synagogue and in the area outside its northwest corner unearthed evidence of one or more structures that predated the synagogue, but the nature of these structures is unknown [26,27].

#### Late medieval synagogue.

**Fig 3:**
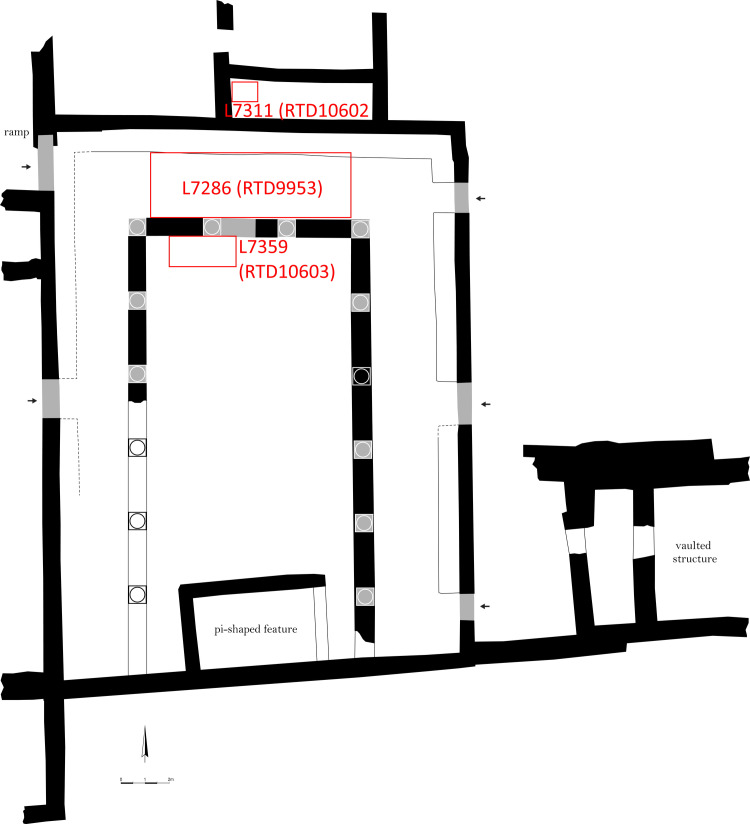
Plan of the late medieval synagogue at Huqoq at the end of the 2019 excavation season. Red boxes show the contexts of radiocarbon samples related to the fill overlying the late Roman mosaic floor, and one sample from outside the synagogue building that is related to a clay installation (L7311) that belongs to an intermediate phase between the late Roman and late medieval synagogues. Plan by Slava Pirsky, with adaptations by Dennis Mizzi and Michael Chazan.

The late medieval synagogue was erected on the same spot as the late Roman synagogue, reusing some of the earlier structure’s architectural elements but expanding it in size (Fig 3) [16]. Specifically, the late medieval building reused the late Roman synagogue’s east wall (but extended it farther to the south) and north wall (but extended it farther to the west). Although the east and north walls are on the line of the original synagogue’s walls, the incorporation of reused ashlar blocks of different sizes bonded by grey mortar indicates that parts were rebuilt in the late medieval period. Like the late Roman synagogue that preceded it, the late medieval building is a basilica but with at least three doorways in the east wall and one in the center of the west wall. The nave is separated from the north, east, and west aisles by a stylobate, which on the north and east consists of meticulously dressed stones that supported columns on pedestals. The north and east stylobates overlie the lines of the corresponding late Roman synagogue stylobates, and the west stylobate overlies the line of the late Roman synagogue’s west wall. The late medieval building’s stylobates, columns, and pedestals appear to have originated in the late Roman synagogue, having been lifted ca. one meter to the level of the late medieval floor above. The late medieval synagogue measures ca. 24 ×  17.86 m. From east to west, the interior of the building spanned ca. 16.80 m. The nave was ca. 8.44 m wide, whereas the west and east aisles—respectively, 2.22 m and 2.66 m wide—were of unequal length.

The late medieval synagogue’s floor is made of a bedding of large cobbles over a thick concrete-like matrix, covered with a thick layer of plaster. Very small patches of white or geometric and floral mosaics are preserved, embedded in the plaster on top of the matrix in the north and east aisles [28]. A single white tessera, embedded in plaster found *in situ* abutting the inside of the north stylobate, indicates that the nave was paved with mosaics as well. The late medieval floor sealed the layers of fill that were deposited on top of the late Roman mosaics.

#### Fill overlying the late Roman synagogue floor.

**Fig 4:**
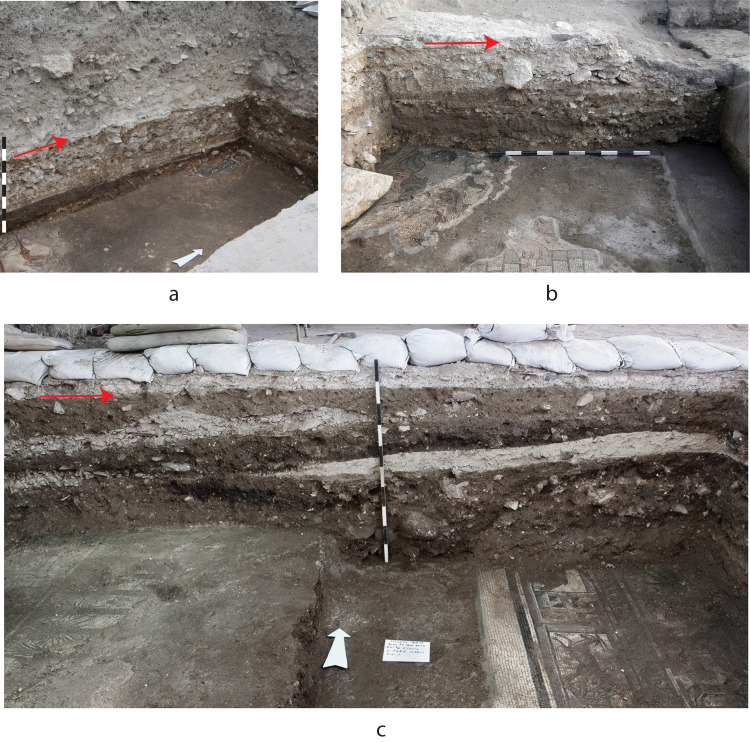
Profiles showing the fill overlying the late Roman mosaic floor. a) east aisle, looking northwest; b) east aisle, looking north; c) west aisle and nave, looking north. Red arrows indicate the make-up of the floor of the late medieval synagogue. Photographs by Jim Haberman.

The sediments overlying the synagogue floor are fill, and micromorphological analysis points to overall homogeneity of sediments comprising silty clay with mixed limestone inclusions and, in some contexts, mineral aggregates (quartz, basalt), phytoliths, charcoal, shell, and bone [1]. Anthropogenic inclusions are absent from the first two centimetres above the late Roman synagogue floor. Dumping events of sediments with differing composition are clearly evident in profiles as shown in Fig 4 and were distinguished during excavation as loci. Inside the late Roman synagogue, the depth of the fill above the mosaic floor varies from ca. 60 cm (east and north aisles) to 80 cm (nave), whereas the fill below the west aisle of the late medieval building, which is located outside the west wall of the late Roman synagogue, was about one meter thick. The total volume of the fill can be calculated as roughly between 200 and 250 cubic meters (including the unexcavated segment of fill in the south part of the late medieval building’s west aisle). The fill represents a very significant component of the labor involved in the construction of the late medieval synagogue. There is no evidence of any living horizons within or at the base of this fill deposit, although in some places a thin accumulation layer of compact, yellowish-white sediment was found covering the late Roman synagogue’s mosaics, mostly in the north and west aisles and at the south end of the east aisle. In the north part of the west aisle, this thin layer (together with the mosaic floor and underlying bedding) was cut by the foundation trench of the late medieval synagogue’s west stylobate. As of 2019, a total of 109,868 ceramic sherds had been recovered from the fill, 6,270 of which are diagnostic and none of which belongs to restorable vessels. Other artifacts recovered from the fill include glass fragments, metal objects, coins, and 26 roof tiles.

## Methods

The Huqoq Excavation Project is directed by Jodi Magness of the University of North Carolina at Chapel Hill under permits granted by the Israel Antiquities Authority (G-19/2011; G-11/2012; G-2/2013; G-16/2014; G-34/2015; G-6/2016; G-24/2017; G-16/2018; G-2/2019; G-4/2022; G-1/2023). All necessary permits were obtained for the described study, which complied with all relevant regulations.

### Radiocarbon dating

The samples for radiocarbon dating discussed here were collected during excavation by the archaeological team and placed directly in aluminum foil envelopes with detailed recording of context. Sample preparation and analysis were carried out at the Dangoor REsearch Accelerator Mass Spectrometer (D-REAMS), a dedicated carbon-only AMS system at the Weizmann Institute of Science (Rehovot, Israel) [29]. Sample preparation procedures at D-REAMS are described in [22]. Dates were calibrated using OxCal software version 4.4 based on the calibration data in IntCal 20 curve [30,31].

## Results

### Radiocarbon ages

**Fig 5:**
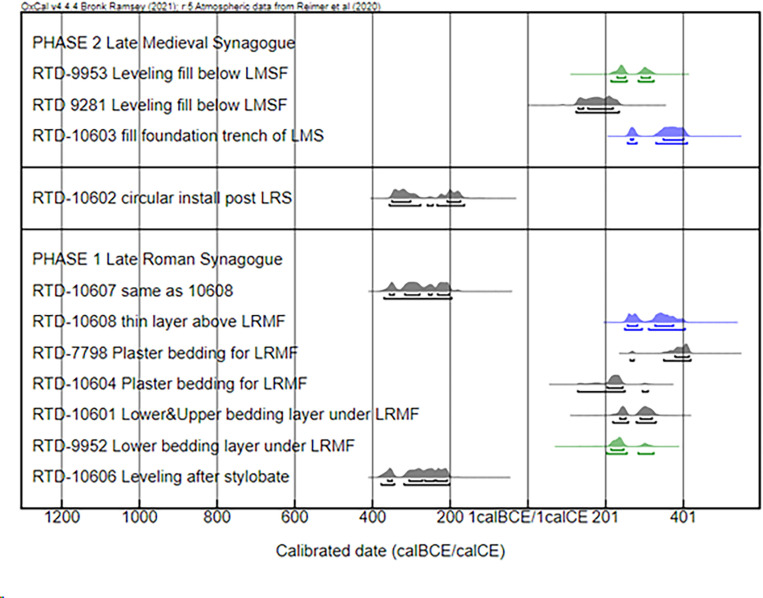
Probability distribution of the radiocarbon calibrated ranges. Colour code: black charcoal, green olive pits, blue seeds. The samples are grouped in two main phases and listed in stratigraphic order with the earlier at the bottom. LRMF Late Roman Mosaic Floor, LMSF Late Medieval Synagogue Floor, LRS Late Roman Synagogue.

Radiocarbon dates are provided by samples from macroscopically identified critical contexts that were submitted for analysis and are grouped into two phases for the purposes of this study: Phase 1 (the late Roman synagogue and its mosaic floor), and Phase 2 (the late medieval synagogue). One sample was collected in 2014, and a set of ten samples was collected in 2018 and 2019. Four samples consisted of charred seeds or olive pits and seven were wood charcoal. After chemical pre-treatment to remove the possible carbon environmental contamination, all samples provided enough material for the AMS analysis. The final carbon percentage in the pre-treated material was above 60% for all the samples indicating a good state of preservation of the charred material. The results are presented in Table 1 and Fig 5 below. The samples come from the following contexts:

**Table 1:** Radiocarbon age determination in BP (Before Present) and calibrated ranges at ± 1σ and ± 2σ for the samples dated.

Lab Number RTD	Field ID	Sample Type	Libby Age year ± 1σ BP	Calibrated range ±1σ (68.3% probability)	Calibrated range ± 2σ (95.4% probability)
**Late Medieval Synagogue (Phase 2)**					
RTD-9953	Huqoq2018 L7286, B71867	Olive Pit	1801 ± 18	231CE (37.0%) 252CE 293CE (31.3%) 315CE	215CE (51.5%) 256CE 285CE (43.9%) 325CE
RTD-9281	Huqoq 2017 L5090, B51302	Charcoal	1861 ± 23	90 CE (7.6%) 100 125 (43.4%) 180CE 190 CE (17.3%) 210 CE	85 CE (95.4%) 225 CE
RTD-10603	Huqoq 2018 L7359, B72763	Seed	1705 ± 21	265CE (7.4%) 272CE 349CE (60.8%) 401CE	258CE (18.8%) 281CE 331CE (76.6%) 411CE
RTD-10602	Huqoq 2018 L7311, B72063	Charcoal	2179 ± 21	350BCE (41.7%) 303BCE 208BCE (26.5%) 174BCE	357BCE (54.4%)277BCE 259BCE (2.2%) 245BCE 234BCE (38.8%) 164BCE
**Late Roman Synagogue (Phase 1)**					
RTD-10607	Huqoq 2018 L7374, B72804	Charcoal	2210 ± 21	357BCE (8.7%) 345BCE 317BCE (30.7%) 279BCE 256BCE (5.5%) 248BCE 233BCE (23.4%) 204BCE	371BCE (95.4%) 197BCE
RTD-10608	Huqoq 2018 L7374, B72805	Seed	1728 ± 21	257CE (24.3%) 283CE 328CE (44.0%) 375CE	250CE (32.0%) 295CE 312CE (63.5%) 405CE
RTD-7798	Huqoq 2014 #2 L3183, B3143	Charcoal	1675 ± 16	380CE (68.3%) 416CE	264AD (5.0%) 274CE 351CE (90.4%) 420CE
RTD-10604	Huqoq 2018 L7369, B72797	Charcoal	1830 ± 20	205CE (68.3%) 245CE	129CE (91.8%) 250CE 296CE (3.7%) 310CE
RTD-10601	Huqoq 2018 L7282, B71945	Charcoal	1790 ± 20	238CE (23.3%) 253CE 290CE (45.0%) 320CE	220CE (36.2%) 259CE 280CE (59.2%) 330CE
RTD-9952	Huqoq 2018 L7297, B72002	Olive Pit	1815 ± 18	215CE (68.3%) 247CE	204CE (75.4%) 256CE 285CE (20.0%) 325CE
RTD-10606	Huqoq 2018 L7390, B72988	Charcoal	2225 ± 20	361BCE (7.9%) 350BC 306BCE (23.0%) 272BCE 266BCE (17.4%) 241BCE 236BCE (20.0%) 208BCE	378BCE (17.5%) 344BCE 319BCE (78.0%) 202BCE
**Disturbed Context**					
RTD-9282	Huqoq 2017 L7228, B71452	Charcoal	1670 ± 32	266CE (3.9%) 272CE 353CE (64.3%) 421CE	257CE (10.7%) 282CE 329CE (79.2%) 436CE 464CE (1.7%) 475CE 500CE (3.9%) 531CE
RTD-9283	Huqoq 2017 L7232, B71538	Charcoal	1255 ± 21	685CE (65.7%) 744CE 794CE (2.5%) 798CE	674CE (77.7%) 779CE 787CE (17.3%) 829CE 860CE (0.4%) 864CE

#### Late Roman synagogue (Phase 1).

RTD-10606 (Locus 7390, Basket 72988): Levelling fill associated with the late Roman synagogue’s construction, which was deposited after the late Roman west stylobate was put into place.

RTD-9952 (Locus 7297, Basket 72002): Lower layer of the bedding underlying the late Roman mosaic floor in the north part of the synagogue’s east aisle; here, the upper layer of the bedding was poorly preserved except in the area surrounding a small patch of surviving mosaic.

RTD-10601 (Locus 7282, Basket 71945): Upper layer of bedding for the late Roman mosaic floor in the northeast corner of the synagogue, which comprised a hard layer of fine plaster with a smooth, flat surface.

RTD-10604 (Locus 7369, Basket 72797): Bedding for the late Roman mosaic in the west aisle of the synagogue. The lower of the two exposed layers comprised white plaster with an inclusion of very small rocks and pebbles (<1–2 cm) and a rock-hard surface. It was topped by an upper, softer layer of white plaster that included many specks of charcoal.

RTD-7798 (Locus 3183, Basket 31436): Plaster bedding under the late Roman mosaic floor.

RTD-10608 (Locus 7374, Basket 72805): Thin accumulation layer of compact, yellowish-white earth directly above the late Roman mosaic floor in the north part of the synagogue’s west aisle.

RTD-10607 (Locus 7374, Basket 72804): See context description for RTD 10608. Note that although these samples come from the same locus, they produced age determinations that are 500 years apart.

#### Late medieval synagogue (Phase 2).

RTD-10603 (Locus 7359, Basket 72763): Upper level of fill in the foundation trench on the south side of the late medieval north stylobate.

RTD-9281 (Locus 5090, B51302): Sample taken directly from standing profile from the leveling fill below the floor of the late medieval synagogue.

RTD-9953 (Locus 7286, Basket 71867): Construction/leveling fill for the floor of the late medieval synagogue’s north aisle.

Another sample (RTD-10602; Locus 7311, B72063) comes from a circular clay installation associated with a layer of ash embedded within a thick fill that sealed the foundations of the north wall of the late Roman synagogue (reused in the late medieval period). Stratigraphically, this locus postdates the construction of the late Roman synagogue and predates structures abutting the late medieval building’s north wall.

Two samples (RTD-9282 and RTD-9283) were collected in 2017 from exposures at the edge of a pit in the northeast corner of the late medieval synagogue, which cut through the building’s floor, the underlying leveling fills, the late Roman mosaic, and further fills below. It subsequently became clear that the sampled material was introduced by the excavation of the pit and thus not *in situ*. These ages are not included in Fig 5 but are included in Table 1 for completeness of reporting.

Table 1 presents the calibrated ranges at ± 1σ (1 standard deviation) and ± 2σ (2 standard deviation), which represent a probability of 68.3% and 95.4%, respectively, that the correct date is included in the given chronological range. In cases where the calibration curve has several wiggles, the full calibrated range is not represented as a single time interval but as several non-contiguous time intervals, and the probability for each one is provided. In the probability distribution plot in Fig 5, the upper bracket below the curve represents a probability level of 68.3%, whereas the lower bracket represents a probability level of 95.4%.

The age determinations do not reflect the stratigraphic relationship between Phases 1 and 2, as for both phases the dates are very similar. Sample RTD-7798, a wood charcoal material, provides the best minimum date or *terminus post quem* for the construction of the late Roman synagogue at ca. 400 calCE. A slightly later date is possible given the old wood effect. The range of RTD-7798 overlaps in the two sigma range with RTD-10608, which is a seed found embedded in the thin accumulation layer of yellowish-white earth directly above the late Roman mosaic floor. It is striking that for the entire group of radiocarbon dates there are no determinations—with the exception of RTD-9283, which is from a disturbed, post-medieval, context—that are later than the beginning of the 5th century.

## Discussion

The dating of archaeological sites in late Roman and Byzantine Galilee (4th – 6th centuries CE) is based largely on the associated coins and pottery. The pottery consists of imported fine wares and local types. The fine wares were manufactured in workshops around the Mediterranean, primarily in North Africa, Asia Minor, and Cyprus. Fine ware types can be dated within relatively narrow time ranges (often one hundred years or less), with changes reflecting the demands and preferences of consumers for new forms and decoration. Hayes’ 1972 publication remains the authoritative reference for these fine wares [32]. However, imported fine wares are much less common at Galilean sites than local products. The local ceramic types of late Roman Galilee were studied by Adan-Bayewitz, who focused primarily on kitchen wares produced in the Kefar Hananya workshop [33]. These types generally cannot be dated more closely than within a range of 200-300 years and ceased to be produced altogether in the 5th century [13]. By the 9th – 10th centuries, most fine wares in the Near East – both imported and locally-produced – were glazed, and even some coarse wares (such as cooking pots) were glazed. Glazed pottery is ubiquitous at sites in Galilee from the medieval period on and can be dated within relatively narrow time ranges as the forms and decoration (e.g., the color of the glazes and other decoration such as incision) changed rapidly [34,35].

Pottery from the foundation trench of the synagogue’s east wall, which was excavated in 2012, provides a *terminus post quem* in the late 4th to early 5th centuries for its construction. The latest closely datable piece of pottery is a Cypriot Red Slip Ware Form 1 bowl rim, a type with a range from ca. 370/380 to the third quarter of the 5th century CE [1,28,33,36,37]. Four coins found in an undisturbed layer of building chips in the same foundation trench date between 323 and 348 CE and include issues of Crispus (323–324 CE), Constans (337–340), Constantius II (337–340), and Constans (347–348). The results of the final excavation season (2023) indicate that the synagogue’s east wall is founded on bedrock, with no traces of an earlier wall at its base.

The latest pottery recovered from the bedding for the mosaic depicting Samson carrying the gate of Gaza consists of local types that range in date from the 2nd to mid-4th centuries CE. Sample RTD-7798, which provides the best minimum date or *terminus post quem* for the construction of the late Roman synagogue at ca. 400 calCE, comes from this same bedding, which was sealed beneath the mosaic floor. Below the bedding were layers of construction fill without any evidence of an earlier floor, although scattered remains of walls predating the late Roman synagogue were unearthed below its north and west aisles and in the area outside its northwest corner. The latest pottery from these fills is the rim of a Kefar Hananya Form 1E bowl, which has a range from the mid-3rd to 5th centuries CE [1,13,28].

In 2016, a 0.80 ×  0.70 m sounding was made at the north end of the nave (north of the Noah’s Ark panel), in a spot where the mosaics were not preserved but the bedding was intact. The bedding consisted of a 0.5 m thick compact grey layer mixed with small pebbles set on top of a layer of cobbles. Inside the bedding was the rim of a Kefar Hananya Form 4D cooking pot, dated from ca. 300 CE to the early 5th century [1,28]. Below the cobbles was a thick layer of fill containing only Hellenistic pottery and the rim of an early Roman conical grooved glass bowl, which presumably derived from dumps associated with earlier periods of occupation at Huqoq or were mixed with residual fills into which the synagogue was built.

A sounding made in 2022–2023 under the floor at the south end of the late Roman synagogue’s west aisle yielded large numbers of tesserae in and below the bedding, suggesting that the mosaic in this part of the building had been repaired or replaced. The latest datable artifacts from this sounding include fragments of northern stamped (buff ware) oil lamps of the 4th –5th centuries [38]. No evidence of significant repairs to the mosaics or discontinuity in the floor were detected anywhere else in the late Roman synagogue.

**Fig 6:**
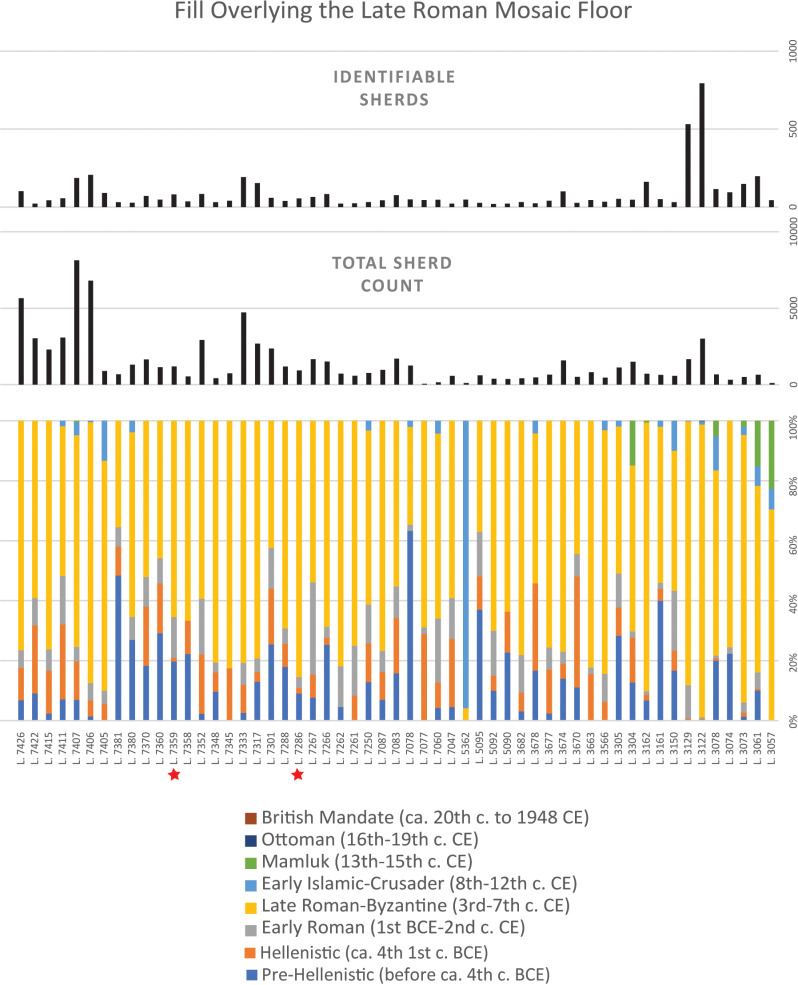
Ceramic assemblages by locus from the fill overlying the late Roman mosaic floor including a breakdown of identifiable sherds by period, the total number of sherds, and the number of identifiable sherds. Only loci with more than 20 identifiable sherds are included. Note that loci are presented in an ascending order that does not reflect the stratigraphic relationship between them. Also note that the loci are not of consistent volume. Red stars indicate loci with associated radiocarbon ages.

**Fig 7:**
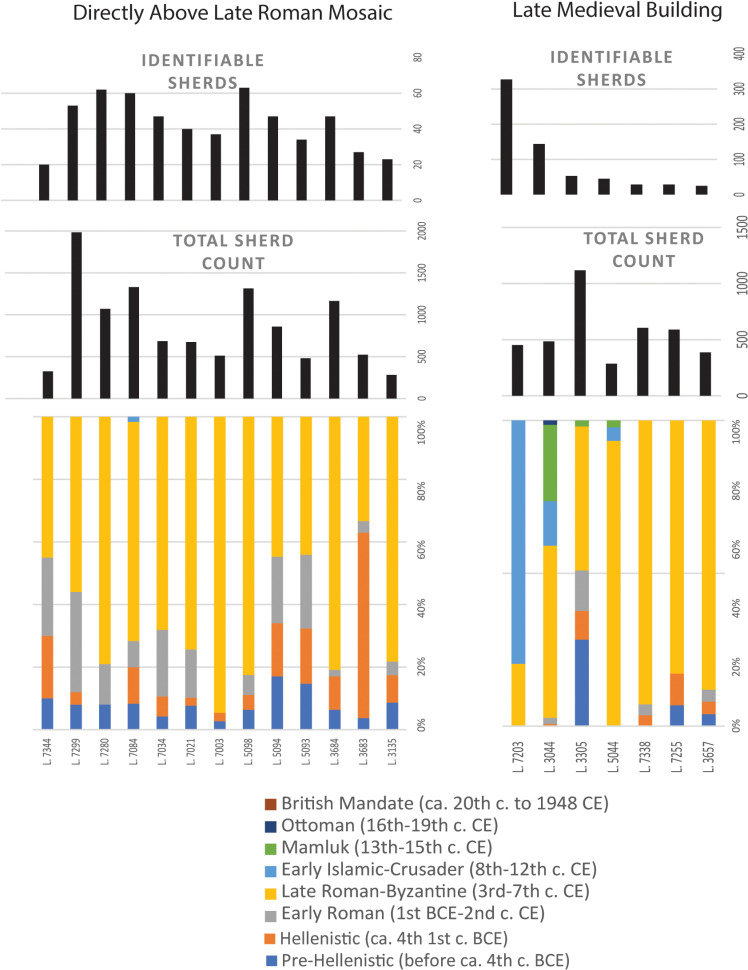
(a) Ceramic assemblages by locus from deposits immediately overlying the late Roman mosaic floor. (b) Ceramic assemblages by locus from the make-up of the floor of the late medieval synagogue, including a breakdown of identifiable sherds by period, the total number of sherds, and the number of identifiable sherds. Only loci with more than 20 identifiable sherds are included. Note that loci are presented in an ascending order that does not reflect the stratigraphic relationship between them. Also note that the loci are not of consistent volume.

The late medieval synagogue’s dating is based on ceramic evidence. Although most of the pottery found in the fills under the building’s floor is late Roman–Byzantine (4th – 6th centuries CE), there are also later types ranging from the 8th through the 13th centuries, and a small number of sherds from the 14th – 15th centuries, which indicate a late medieval date for the building (Figs 6 and 7) [1,16]. At some point in the 15th century, the late medieval synagogue was remodeled, and in the following centuries partition walls were installed, silos and pits were dug into the floor, and elements of the building were robbed out.

The results of the radiocarbon analysis conform with the pottery data to place the construction of the late Roman synagogue at the end of the 4th century or beginning of the 5th century CE. The single age of ca. 400 CE from a sample taken from the construction material for the late Roman mosaic floor provides a minimum date (RTD-7798). It is important to emphasize that this sample was embedded in the plaster bedding of the floor and thus cannot be derived from the use-life of the synagogue. The earlier ages associated with samples from under the mosaic floor are best interpreted as a result of the reuse of materials from existing deposits at the time of the construction of the late Roman synagogue, as amply illustrated by the ceramic evidence. Furthermore, except for the panel at the south end of the west aisle, there are no signs of significant repairs to the mosaics nor any indication that the various mosaic panels were installed at different times. The same phenomenon is reflected also in the construction of the late medieval synagogue, the builders of which recycled much earlier material. This explains the similar distribution of radiocarbon dates across Phases 1 and 2 and the preponderance of late Roman – Byzantine pottery in the late medieval synagogue’s construction fills.

There is a clear difference in depositional processes between the monumental synagogue at the core of the settlement and the domestic areas surrounding it. Our ongoing study of the pottery and coins from domestic units in the nearby ancient village indicates there was a series of occupation phases, separated by brief gaps, starting from the late 4th or early 5th centuries to the 13th – 15th centuries. In contrast, as noted above (see Site Architecture and Stratigraphy), there appears to be a depositional gap in the synagogue area between ca. 400 and 1400 CE, although the fills below the late medieval floor contain small quantities of Byzantine, early Islamic, Crusader, and Mamluk pottery. Because of this depositional gap, the ages of the radiocarbon samples from these fills provide no chronological control over the use-life of the late Roman synagogue. The pottery and radiocarbon data are consistent with the interpretation of this fill as having been derived partly from the same deposits used in the initial construction of the late Roman synagogue and partly from the ancient village’s middens. This interpretation is supported also by the micromorphological analysis of these deposits [1]. The use-life of the synagogue, and, indeed, the entire interval between the two phases of construction, are a striking depositional lacuna. It thus appears that after the late Roman synagogue went out of use and was abandoned, it was cleared out and left empty for centuries (with the mosaics still exposed), although at some point parts of the superstructure collapsed, causing damage to sections of the mosaics. When the synagogue was reconstructed in the late medieval period, the collapsed architectural pieces were cleared out and 0.6-0.8 m of construction fills were dumped on top of the mosaics to raise the floor to its new level [16]. Although the late Roman synagogue was a monumental structure in the heart of the village, the activities related to its use-life and subsequent abandonment are not preserved.

## Conclusion

The radiocarbon dates indicate clearly that the late Roman synagogue was built around the beginning of the 5^th^ century CE. This is supported by the age of the RTD 7798 sample as well as by the samples in the bedding material below the mosaic floor, which date to the 2nd and 3rd centuries CE. Apparently, the material used for the fill under the mosaic floor derives largely from late Roman deposits, pointing to intense activity during this period. The radiocarbon calibration curve for this period has steep slopes and some brief plateaus that could be resolved with more short-lived dates from secure contexts. Sample RTD-7798 provides the best minimum age or *terminus post quem* in this regard, and thus corroborates the dating of the Huqoq synagogue based on ceramic data. In view of the ongoing debate about the chronology of Galilean-type synagogues, alternative methods of dating such as radiocarbon, which is not commonly employed in late antique contexts in Levantine archaeology, could therefore help break the impasse.

In the case of Huqoq, the use of fill derived from materials predating the late Roman synagogue’s construction results in the absence of a stratigraphic sequence that can be established to constrain calibrated ages. These results point to the importance of detailed contextual data to resolve the chronology of monumental religious architecture in the late Roman and Byzantine world.
